# Comparing HLA Shared Epitopes in French Caucasian Patients with Scleroderma

**DOI:** 10.1371/journal.pone.0036870

**Published:** 2012-05-15

**Authors:** Doua F. Azzouz, Justyna M. Rak, Isabelle Fajardy, Yannick Allanore, Kiet Phong Tiev, Dominique Farge-Bancel, Marielle Martin, Sami B. Kanaan, Philippe P. Pagni, Eric Hachulla, Jean Robert Harlé, Rémi Didelot, Brigitte Granel, Jean Cabane, Jean Roudier, Nathalie C. Lambert

**Affiliations:** 1 Laboratoire d’Immunogénétique de la Polyarthrite Rhumatoïde, INSERM UMRs1097, Marseille, France; 2 Service de Médecine Interne, Centre National de Référence de la Sclérodermie Systémique, Hôpital Claude Huriez, Lille, France; 3 Université Paris Descartes, Service de Rhumatologie A, Hôpital Cochin, Paris, France; 4 Hôpital Cochin, Paris, France; 5 Service de Médecine Interne, Hôpital St Antoine, Paris, France; 6 Service de Médecine Interne et Pathologie Vasculaire, Hôpital St Louis, Paris, France; 7 Hôpital St Louis, Paris, France; 8 Service de Médecine Interne, Hôpital La Conception, Marseille, France; 9 Centre d’Examen de Santé Assurance Maladie, Marseille, France; 10 Service de Médecine Interne, Hôpital Nord, Marseille, France; 11 Service de Rhumatologie, Hôpital Ste Marguerite, Marseille, France; University of Cape Town, South Africa

## Abstract

Although many studies have analyzed HLA allele frequencies in several ethnic groups in patients with scleroderma (SSc), none has been done in French Caucasian patients and none has evaluated which one of the common amino acid sequences, ^67^FLEDR^71^, shared by HLA-DRB susceptibility alleles, or ^71^TRAELDT^77^, shared by HLA-DQB1 susceptibility alleles in SSc, was the most important to develop the disease. HLA-DRB and DQB typing was performed for a total of 468 healthy controls and 282 patients with SSc allowing FLEDR and TRAELDT analyses. Results were stratified according to patient’s clinical subtypes and autoantibody status. Moreover, standardized HLA-DRß1 and DRß5 reverse transcriptase Taqman PCR assays were developed to quantify ß1 and ß5 mRNA in 20 subjects with HLA-DRB1*15 and/or DRB1*11 haplotypes. FLEDR motif is highly associated with diffuse SSc (χ^2^ = 28.4, p<10−6) and with anti-topoisomerase antibody (ATA) production (χ^2^ = 43.9, p<10−9) whereas TRAELDT association is weaker in both subgroups (χ^2^ = 7.2, p = 0.027 and χ^2^ = 14.6, p = 0.0007 respectively). Moreover, FLEDR motif- association among patients with diffuse SSc remains significant only in ATA subgroup. The risk to develop ATA positive SSc is higher with double dose FLEDR than single dose with respectively, adjusted standardised residuals of 5.1 and 2.6. The increase in FLEDR motif is mostly due to the higher frequency of HLA-DRB1*11 and DRB1*15 haplotypes. Furthermore, FLEDR is always carried by the most abundantly expressed ß chain: ß1 in HLA DRB1*11 haplotypes and ß5 in HLA-DRB1*15 haplotypes.

In French Caucasian patients with SSc, FLEDR is the main presenting motif influencing ATA production in dcSSc. These results open a new field of potential therapeutic applications to interact with the FLEDR peptide binding groove and prevent ATA production, a hallmark of severity in SSc.

## Introduction

Systemic Sclerosis (SSc) or Scleroderma, is a chronic autoimmune disease with unknown aetiology, characterized by fibrosis, vascular alterations and autoantibodies. Scleroderma is stratified by clinical criteria into two subtypes: limited cutaneous SSc (lcSSc) mainly affecting the hands, arms and face, and diffuse cutaneous scleroderma (dcSSc), affecting a large area of the skin, at increased risk of cardiac disease, interstitial lung disease, renal crisis and early death. Specific autoantibodies correlate with subtypes. Anti-centromere antibodies (ACA) and anti-topoisomerase antibodies (ATA) are respectively a hallmark of lcSSc and dcSSc, although not always detected and sometimes observed in each other group. Other autoantibodies, such as anti-RNA polymerase, antifibrilllin (AFA) or anti-U3RNP associate with particular clinical manifestations [1], [2], [3], [4].

Disease subtypes and auto-antibody profiles are strongly associated with HLA-DRB and DQB alleles. The most frequently reported associations with dcSSc are HLA-DRB1*11, HLA-DQB1*03 among European and North American Caucasians and Africans [3], [5], [6]. However autoantibody specificities are different depending upon the HLA-DRB1*11 allele: HLA-DRB1*11∶04 is associated with ATA in Caucasians and HLA-DRB1*1101 with AFA in Africans. HLA-DRB1*15∶02- HLA-DQB1*06∶01 haplotypes are commonly found among Asians with dcSSc and ATA [7]. Less commonly mentioned are associations of HLA-DRB1*0802 with dcSSc in Japanese and HLA-DRB1*0804 with ATA positive patients in American blacks [3], [7], [8].

Most common HLA-DR associated with dcSSc (HLA-DRB1* 11∶01, *11∶04, *15∶01, *08∶02…) have in common an amino acid sequence ^67^FLEDR^71^ on their ß chain (**Table 1**). Similarly, most common HLA-DQB1 susceptibility alleles, DQB1*03∶01, *03∶02, *04∶01, *04∶02, *06∶01 and *06∶02, code for a common ^71^TRAELDT^ 77^ motif on their ß chain (**Table 2**). Reveille et al. have proposed that additionally to TRAELDT, an amino acid, tyrosine at position 30 (^30^Y) present on HLA-DQB1*03 and DQB1*06∶01-:03 and *6∶05-:07 alleles (see **Table 2**) strengthens the association of ATA with Caucasians and African Americans [9].

**Table 1:** Shared amino acid sequences of the most common DRß chains.

DRB1 alleles	Amino acid number				
	67	68	69	70	71
*01∶01, *01∶02, *04∶03–08, *14∶02	L	L	E	Q	R
*01∶03, *04∶02, *11∶02, *13∶01, *13∶02, *13∶04	I	–	–	D	E
*15∶01-:03	I	–	–	–	A
*15∶04	F	–	–	–	A
*16∶01, *16∶03, *16∶04, *16∶08	***F***	–	–	***D***	–
*16∶02, *14∶03	–	–	–	D	–
*16∶05, *16∶07, *12∶01, *12∶03–05, *07∶01,*0703, 0803	I	–	–	D	–
*03∶01–03∶11, *04∶01	–	–	–	–	K
*11∶01, *11∶04 **–** 06, *11∶09	***F***	–	–	***D***	–
*11∶03	F	–	–	D	E
*11∶07	–	–	–	–	K
*12∶02, *13∶05	***F***	–	–	***D***	–
*13∶03	I	–	–	D	K
*14∶01, *14∶04	–	–	–	R	–
*08∶01, *08∶02, *08∶04 **–** ***09***	***F***	–	–	***D***	–
*09∶01	F	–	–	R	–
*10∶01	–	–	–	R	–
**DRB3 alleles**					
*01∶01–03, *02∶01–08, *03∶01–03	–	–	–	–	K
**DRB4 alleles**					
*01∶01-:05	–	–	–	R	–
**DRB5 alleles**					
*01∶01,*01∶02,*01∶04,*01∶05	***F***	–	–	***D***	–
*01∶03	–	–	–	D	T
*01∶06,*02∶02,*02∶03	I	–	–	–	A
*01∶07	I	–	–	D	–
*01∶09	F	–	–	N	–
*02∶04	F	–	–	–	A

**Table 2:** Shared amino acid sequences of the most common HLA-DQß chains.

	Amino acid number								
DQB1 alleles	30	…	71	72	73	74	75	76	77
*05∶01, *05∶02, *05∶03	H	…	A	R	A	S	V	D	R
*05∶04	***Y***	…	D	–	–	–	–	–	
*03∶01-*03∶04,*06∶01/2/5/7/9	***Y***	***…***	***T***	**–**	**–**	***E***	***L***	**–**	***T***
*06∶03, 06∶04	***H***	***…***	***T***	**–**	**–**	***E***	***L***	**–**	***T***
*02∶01	S	…	K	–	–	A	–	–	–
*03∶05	***Y***	***…***	***T***	**–**	**–**	***E***	***L***	**–**	***T***
*04∶01, *04∶02	***Y***	…	D	–	–	–	–	–	T

HLA-DR molecules are heterodimers composed of a non-polymorphic α chain, encoded by DRA and a highly polymorphic ß chain (ß1 to ß5) encoded by DRB1*, DRB3*, DRB4* or DRB5* genes. In HLA-DR15 molecules, the α chain associates either with a ß1 or ß5 chain coded respectively by DRB1*15 or DRB5*01. The ^67^FLEDR^71^ motif is only expressed on the ß5 chain. Conversely, in HLA-DR11 molecules, the ^67^FLEDR^71^ motif is expressed on the ß1 chain encoded by DRB1*11 and not on the co-expressed ß3chain encoded by DRB3*02. Consequently, two heterodimeric HLA-DR molecules are co- expressed at the cell surface. For HLA-DR15, α chain associates with ß1 or ß5 and for DR11, α chain associates with ß1 or ß3. But often only heterodimers with ß1 chains are taken into consideration for antigen presentation purpose, as other chains are often considered as accessory chains. If indeed protein expression for ß1 chains is higher, a person carrying the FLEDR shared epitope on the HLA-DR15 haplotype would have less expression and a deficient FLEDR restricted antigen presentation compared to a person carrying it on the HLA-DR11 haplotype.

In the current study, we evaluated for the first time the relative risk conferred by one or two ^67^FLEDR^71^ and/or one or two ^71^TRAELDT^77^ motives among our Caucasian French patients with SSc. As we know, from previous studies, classification by clinical subtypes and autoantibody profiles could influence results, we conducted a complete analysis with both classifications to determine whether FLEDR and/or TRAELDT motives influence susceptibility to the limited or diffuse form of SSc and/or to autoantibody subsets. We further evaluated the importance of the ß chain carrying the FLEDR shared epitope by quantifying levels of ß chain transcripts.

## Methods

### Patients and Controls

We included 282 Caucasian patients with SSc with no overlapping disease, all with defined SSc type (94 diffuse SSc; 188 limited SSc) and auto-antibody status known for 243 of them (80 without ATA or ACA, 89 with ACA and 74 with ATA). Patients were enrolled in collaboration with 7 French hospitals from Paris, Marseille and Lille and fulfilled the criteria of LeRoy for SSc [10]. Altogether 235 women and 47 men were analysed for HLA-DR and DQ allele frequencies. Mean age at diagnosis was 49.3 years +14.3 (mean ± SD).

In parallel, 468 healthy Caucasian controls were recruited at the Centre d’Examen de Santé de l’Assurance Maladie (CESAM), Marseille, France (N = 154, mean age at the inclusion was 52.5 years ± 7.5 [mean ± SD]) and at Claude Huriez Hospital in Lille, France (N = 314, mean age at inclusion was 35.4 years ± 10.2 [mean ± SD]). None of the controls had any symptom or familial history of autoimmune disorder.

### Ethics Statements

Controls from Marseille are registered at INSERM under the Biomedical Research Protocol number RBM-04-10.

Controls from Lille were drawn from a DNA bank created in the biological laboratory in 1993. Written consent forms obtained according to the Declaration of Helsinki were signed [11].

### HLA-DRB1 and DQB1 Typing

Genomic DNA was extracted from peripheral blood by standard methods and HLA genotyping was performed either by using PCR-RFLP, as previously described for samples received in Lille or sequence-specific oligonucleotide (SSO) HLA-DRB1 and HLA-DQB1 typing kit, (RELI SSO, Dynal, Invitrogen, Bromborough, Wirral, UK) according to manufacturer’s protocol as previously described for samples received in Marseille [12], [13]. Allelic typing for DRB1 was done at Etablissement Français du Sang (EFS, Marseille, France) for samples received in Marseille.

### RNA Preparation and cDNA Synthesis

Total RNA was extracted from PBMC using GenElute Mammalian total RNA Miniprep Kit (Sigma-Aldrich, St Louis MO, USA). For each RNA sample, DNase I digestion was included as recommended (Sigma-Aldrich, St Louis MO, USA). Concentration of RNA was spectrophotometrically measured and quality was ascertained with Agilent Analyser (Agilent RNA 6000 Nano Kit, Agilent Technologies, Germany). Total RNA was reverse-transcribed using Enhanced Avian HS RT-PCR kit (Sigma-Aldrich, St Louis MO, USA) according to manufacturer’s recommendations.

### Quantitative Comparison of mRNA from HLA-DRB1*15/HLA-DRB5*01 and HLA-DRB1*11/HLA-DRB3*02

For HLA-DRB1*15/DRB5*01 assays, we used previously described real-time PCR assay from Prat et al. with some modifications in primers sequences [14]. For HLA-DRB1*15 assay, the forward primer was modified: 5′-GCTCCCCACTGGCTTTGT-3′, a new reverse primer and probe were designed: 5′-GGCTGTTCCAGTACTCAGCG-3′ and 5′FAM- ACCACGTTTCCTGTGGCAGCCTAAGAG-TAMRA3′, respectively. For HLA-DRB5*01 assays, only the forward primer was modified: 5′- TGGAGGTTCCTACATGGCAA-3′.

We designed HLA-DRB1*11 and HLA-DRB3*02 assays. To ensure that genomic DNA was not amplified, forward primers for each assay were designed to span Exon 1-Exon 2 borders. HLA-DRB1*11 forward primer, reverse primer and probe had respectively the following sequences: 5′-GGACACCAGACCACGTTTCT-3′, 5′-CGCACGTACTCCTCTTGGTTATA-3′ and 5′FAM-ATTGAAGAAATGACACTCAGACGTAGAGTACTCC-TAMRA3′. HLA-DRB3*02 primers and probe design was as follow: forward primer 5′-GGACACCCGACCACGT-3′, reverse primer 5′-CGCGTACTCCTCCTGGTTAT-3′ and probe 5′FAM-CTTGGAGCTGCTTAAGTCTGAGTGTCATTTC-TAMRA3′.

As quantitative comparisons between mRNAs are not trustable since no perfect gene reference exist, we cloned PCR products from each assay (DRB1*15, DRB5*01, DRB1*11 or DRB3*02) in pCR4-TOPO plasmids (pCR4-TOPO TA Cloning Kit, Invitrogen, Carlsbad, CA) to calibrate standards curves from these constructions, as explained below. After bacterial transformation and plasmid extraction (QIAprep Spin Miniprep kit, Qiagen), each construct was checked by sequencing (Cogenics, Grenoble France). Plasmids were then linearized with NcoI restriction enzyme (Invitrogen, Carlsbad, CA) and concentrations for the 4 constructs were adjusted by using a common set of primers and probe specific to the plasmid sequence and designed as follows : forward primer 5′-CAGAATTAACCCTCACTAAAGGGACT-3′, reverse primer: 5′-ATAGGGCGAATTGAATTTAGCG-3′ and probe 5′FAM-TCCTGCAGGTTTAAACGAATTCGCC-TAMRA3′. The 4 standards were then serially diluted. Normalization of concentrations between the 4 constructs was possible because inserts were very similar in size (210 bp and 193 bp for HLA-DRB1*15 and –DRB5*01 respectively and 114 bp and 111 bp for HLA-DRB1*11 and –DRB3*02 respectively). Standards were run with their own set of primers and probes. Adjustment for PCR efficiency of each curve was done and relative comparison between quantities of HLA-DRB1*15/DRB5*01 mRNA and HLA-DRB1*11/DRB3*02 mRNA was calculated.

### Statistical Analysis

Pearson chi-square tests with the adjusted standardized residual method [15] were used to compare frequencies and give an indication of the strength of the association for each shared epitope (FLEDR and TRAELDT) between different groups. Indeed the χ2 test indicates whether there is an association between two categorical variables. However, it does not in itself give an indication of the strength of the association. In order to identify the cells (one dose, two doses…) that have the larger differences between the observed and expected frequencies, we used the adjusted standardized residuals. These differences are referred to as residuals, and they can be standardized and adjusted to follow a Normal distribution with mean 0 and standard deviation 1 [2]. The adjusted standardized residuals, dij, are given by: 
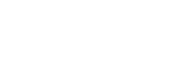
.

Where *Oij* is the observed frequency in the cell in row *i* and column *j* and *Eij* is the expected frequency in the cell in row *i* and column *j*, where *ni* is the total frequency for row *i*, *nj* is the total frequency for column *j*, and *N* is the overall total frequency. The larger the absolute value of the residual, the larger the difference between the observed and expected frequencies, therefore the more significant the association between the two variables.

Adjusted standardized residual >1.96 indicates that the number of cases in that cell is significantly larger than would be expected if the null hypothesis were true, with a significance level of 0.05. An adjusted residual < −2.0 indicates that the number of cases in that cell is significantly smaller than would be expected if the null hypothesis were true.

## Results

### FLEDR Motif is Highly Associated with dcSSc (Table 3)

**Table 3:** Prevalence of FLEDR and TRAELDT in patients with SSc divided by clinical subtypes.

		DcSSc			LcSSc			healthy controls		
		N = 94			N = 188			N = 468		
Motif	Doses	#	%	Adj. Std Resd^a^	#	%	Adj. Std Resd^a^	#	%	Adj. Std Resd^a^
**FLEDR** b	**2**	21	22.3	**3.7**	21	11.2	0.0	42	9.0	*−2.5*
	**1**	50	53.2	**2.3**	84	44.7	0.7	184	39.3	*−2.2*
	**0**	23	24.5	*−4.6*	83	44.1	*−*0.7	242	51.7	**3.8**
**TRAELDT** c	**2**	44	46.8	**2.9**	54	28.7	*−*1.7	155	33.1	*−*0.5
	**1**	42	44.7	*−*1.4	101	53.7	0.7	244	52.1	0.4
	**0**	8	8.5	*−*1.8	33	17.6	1.3	69	14.7	0.1

a adjusted standardized residual >1.96 indicates that the number of cases in that cell is significantly larger than would be expected if the null hypothesis were true (represented in bold), with a significance level of.05. An adjusted residual < −2.0 indicates that the number of cases in that cell is significantly smaller than would be expected if the null hypothesis were true (represented in italic).

b when comparing different subgroups for FLEDR association: DcSSc/LcSSc/healthy controls: χ^2^ = 29.1, p<10^−5^; LcSSc/healthy controls: not significant; DcSSc/healthy controls: χ^2^ = 28.4, p<10^−6^.

c when comparing different subgroups for TRAELDT association: DcSSc/LcSSc/healthy controls: χ^2^ = 10.2, p = 0.029; LcSSc/healthy controls: not significant; DcSSc/healthy controls: χ^2^ = 7.2, p = 0.027.

HLA-DRB1 allelic typing was performed on 468 controls and 282 patients with SSc (94 patients with dcSSc and 188 patients with lcSSc) to identify the FLEDR motif coded by some HLA-DRB alleles (**Table 1** **and Table S1**). Similarly HLA-DQB1 allelic typing was performed on the same number of controls and patients to identify the TRAELDT motif coded by some HLA-DQB1 alleles and the amino acid present at position 30 (**Table 2** **and Table S2**). The presence of FLEDR or TRAELDT on one haplotype is noted: 1 dose, on both haplotypes: 2 doses and on none: 0 dose.

When patients are divided by clinical subtypes (**Table 3**), standardized adjusted residuals give an indication of association for each motif, as explained above. Presence of 2 doses of FLEDR is the most significant risk to develop dcSSc compared to a single dose (respective adjusted standardized residuals: 3.7 and 2.3, see Methods). For TRAELDT having 2 doses is also the most significant risk to develop dcSSc compared to a single dose (respective adjusted standardized residuals: 2.9 and −1.4).

However if we compare which one of the two motives is associated with the highest risk to develop dcSSc, FLEDR motif is at higher risk than TRAELDT (respectively χ^2^ = 28.4, p<10^−6^ and χ^2^ = 7.2, p = 0.027).

The increase of FLEDR motif in patients with dcSSc is mostly due to the higher frequency of HLA-DRB1*11 and DRB1*15 alleles (**Table S1**).

Although TRAELDT motif is increased among patients with dcSSc, none of the TRAELDT positive alleles (HLA-DQB1*03 and *06) are statistically increased (**Table S2**).

As it had been previously proposed that tyrosine at position 30 (^30^Y) could strengthen the TRAELDT association, we analysed this possibility. Indeed, the ^30^Y residue added to TRAELDT slightly strengthens the TRAELDT association with dcSSc (χ^2^ = 10.5, p = 0.0015, data not shown) but remains weaker than FLEDR association (χ^2^ = 28.4, p<10^−6^).

### FLEDR Motif is Highly Associated with Patients with ATA

Patients were divided in three subgroups according to their autoantibody status (**Table 4** **, Tables S3 and S4**): patients without ACA or ATA (Abneg); patients with ACA (ACApos); patients with ATA (ATApos).

**Table 4:** Prevalence of FLEDR and TRAELDT in patients with SSc divided by antibody status.

		SSc Ab neg			SSc ACA pos			SSc ATA pos			Healthy controls		
		N = 80			N = 89			N = 74			N = 468		
Motif	Doses	#	%	Adj. Std Resd^a^	#	%	Adj. Std Resd^a^	#	%	Adj. Std Resd^a^	#	%	Adj. Std Resd^a^
**FLEDR** b	**2**	7	8.8	−0.7	8	9.0	−0.6	21	28.4	**5.1**	42	9.0	−*2.4*
	**1**	41	51.3	1.6	37	41.6	−0.2	42	56.8	**2.6**	184	39.3	−*2.6*
	**0**	32	40.0	−1.2	44	49.4	0.6	11	14.9	−*5.7*	242	51.7	**4.0**
**TRAELDT** c	**2**	29	36.3	0.5	18	20.2	−*2.9*	39	52.7	**3.6**	155	33.1	−0.6
	**1**	36	45.0	−1.1	50	56.2	1.0	33	44.6	−1.2	244	52.1	0.8
	**0**	15	18.8	1.0	21	23.6	**2.4**	2	2.7	−*3.1*	69	14.7	−0.4

a adjusted standardized residual >1.96 indicates that the number of cases in that cell is significantly larger than would be expected if the null hypothesis were true (represented in bold), with a significance level of.05. An adjusted residual < −2.0 indicates that the number of cases in that cell is significantly smaller than would be expected if the null hypothesis were true (represented in italic).

b when comparing different subgroups for FLEDR association: Ab neg/ACA pos/ATA pos/healthy controls: χ^2^ = 48.5, p<10^−8^; Ab neg/healthy controls: not significant; ACA pos/healthy controls: χ^2^ = 0.17, p = 0.9; ATA pos: χ^2^ = 43.9, p<10^−9^.

c when comparing different subgroups for TRAELDT association: Ab neg/ACA pos/ATA pos/healthy controls: χ^2^ = 27.6, p = 0.00013; Ab neg/healthy controls: not significant; ACA pos: χ^2^ = 7.9, p = 0.02; ATA pos/healthy controls: χ^2^ = 14.6, p = 0.0007.

FLEDR motif is highly associated with ATA positive patients (χ^2^ = 43.9, p<10^−9^) whereas TRAELDT has a weaker association (χ^2^ = 14.6, p<10^−3^, **Table 4**).

Double dose of FLEDR motif is significantly increased among patients with ATA (28.4%) compared with healthy controls (9.0%). The risk to develop ATA positive SSc is higher when double dose FLEDR is present (adjusted standardized residuals: 5.1) than when only one dose is present (adjusted standardised residuals: 2.6). The increase of FLEDR motif in patients with ATA is, like for patients with dcSSc, mostly due to the higher frequency of HLA-DRB1*11 and DRB1*15 alleles (**Table S3**). The risk to develop ATA positive SSc is higher when double dose TRAELDT is present (adjusted standardized residuals: 3.6) than when only one dose is present (adjusted standardised residuals: −1.2). The increase of TRAELDT motif in patients with ATA is mostly due to the higher frequency of HLA-DQB1*03 alleles (**Supplementary Table S4**).

Again, the ^30^Y residue added to TRAELDT slightly strengthens the TRAELDT association with ATA positive SSc (χ^2^ = 19.7, p = 0.00005, data not shown) without being higher than FLEDR association (χ^2^ = 43.9, p<10^−9^).

### Prevalence of FLEDR in ATA Positive dcSSc and Not ATA Negative dcSSc

Although ATA is a hallmark of dcSSc, not all patients with dcSSc have ATA, we wondered whether the FLEDR association was mostly with the clinical subtype (dcSSc ATA+ or −) or the autoantibody profile (**Table 5**). Among the 94 patients with dcSSc we were able to obtain information for autoantibody status for 85 who divided into 52 ATA positive and 33 ATA negative. When both groups (dcSSc ATA pos and dcSSc ATA neg) are compared to healthy controls, the only remaining association is with the group positive for ATA. Again FLEDR is the most prevalent shared epitope in this group (χ^2^ = 35.2, p<10^−7^) compared with TRAELDT (χ^2^ = 9.4, p = 0.009). For both motives, having 2 doses confers the highest risk to develop dcSSc with ATA as they have the highest standardized adjusted residuals (respectively for FLEDR and TRAELDT: 4.8 and 2.6).

**Table 5:** Prevalence of FLEDR and TRAELDT in patients with dcSSc divided by antibody status.

		DcSSc ATA pos			DcSSc ATA neg			Healthy controls		
		N = 52			N = 33			N = 468		
Motif	Doses	#	%	Adj.Std Resd^a^	#	%	Adj.Std Resd^a^	#	%	Adj.Std Resd^a^
**FLEDR**	**2**	16	30.8	**4.8**	3	9.1	−0.4	42	9.0	−*3.6*
	**1**	28	53.8	1.9	18	54.5	1.6	184	39.3	−*2.5*
	**0**	8	15.4	−*4.9*	12	36.4	−1.3	242	51.7	**4.8**
**TRAELDT**	**2**	27	51.9	**2.6**	13	39.4	0.5	155	33.1	−*2.5*
	**1**	23	44.2	−1.1	16	48.5	−0.3	244	52.1	1.1
	**0**	2	3.8	−*2.1*	4	12.1	−0.2	69	14.7	1.9

a adjusted standardized residual >1.96 indicates that the number of cases in that cell is significantly larger than would be expected if the null hypothesis were true (represented in bold), with a significance level of.05. An adjusted residual < −2.0 indicates that the number of cases in that cell is significantly smaller than would be expected if the null hypothesis were true (represented in italic).

b when comparing different subgroups for FLEDR association: dcSSc ATA pos/dcSSc ATA neg/healthy controls: Fisher’s exact test p<10^−6^; dcSSc ATA pos/healthy controls: χ^2^ = 35.2, p<10^−7^; dcSSc ATA neg/healthy controls: not significant.

c when comparing different subgroups for TRAELDT association: dcSSc ATA pos/dcSSc ATA neg/healthy controls: Fisher’s exact test p = 0.043; dcSSc ATA pos/healthy controls: χ^2^ = 9.4, p = 0.009; dcSSc ATA neg/healthy controls: not significant.

The ^30^Y residue added to TRAELDT slightly strengthens the TRAELDT association with ATA positive dcSSc (χ^2^ = 13.4, p = 0.0012, data not shown) without being higher than FLEDR association (χ^2^ = 35.2, p<10^−7^).

### TRAELDT Association is Not Solely due to Linkage Disequilibrium with FLEDR

DRB1*11 alleles (generally “FLEDR positive” except DRB1*11∶02 and *11∶03) are in linkage disequilibrium (LD) with DQB1*03∶01 (TRAELDT positive) and HLA-DRB1*15∶01 alleles (FLEDR positive) in LD with HLA-DQB1*06∶02 (TRAELDT positive). Therefore one could argue that the TRAELDT association observed is only due to LD with FLEDR.

However TRAELDT alone is statistically associated with dcSSc and ATA positive individuals as the frequency of individuals negative for FLEDR (0 dose) but positive (1 or 2 doses) for TRAELDT is statistically higher in patients with dcSSc (χ^2^ = 12.9, p = 0.0003) and in patients with ATA (χ^2^ = 19.4, p = 0.000013) compared to controls (**Tables S5 and S6**). This last result indicates TRAELDT has its own contribution to disease susceptibility and autoantibody specificity.

### Increased mRNAs of Beta Chains with FLEDR Motif in Patients and Controls

The HLA-DRB1*1104/DRB1*1501 genotype was present in 7 out of 19 patients with ATA positive dcSSc with double dose FLEDR but rarely seen in healthy controls with double dose FLEDR (3/42, 2 tailed Fisher’s test, p<0.007, data not shown).

**Figure 1:**
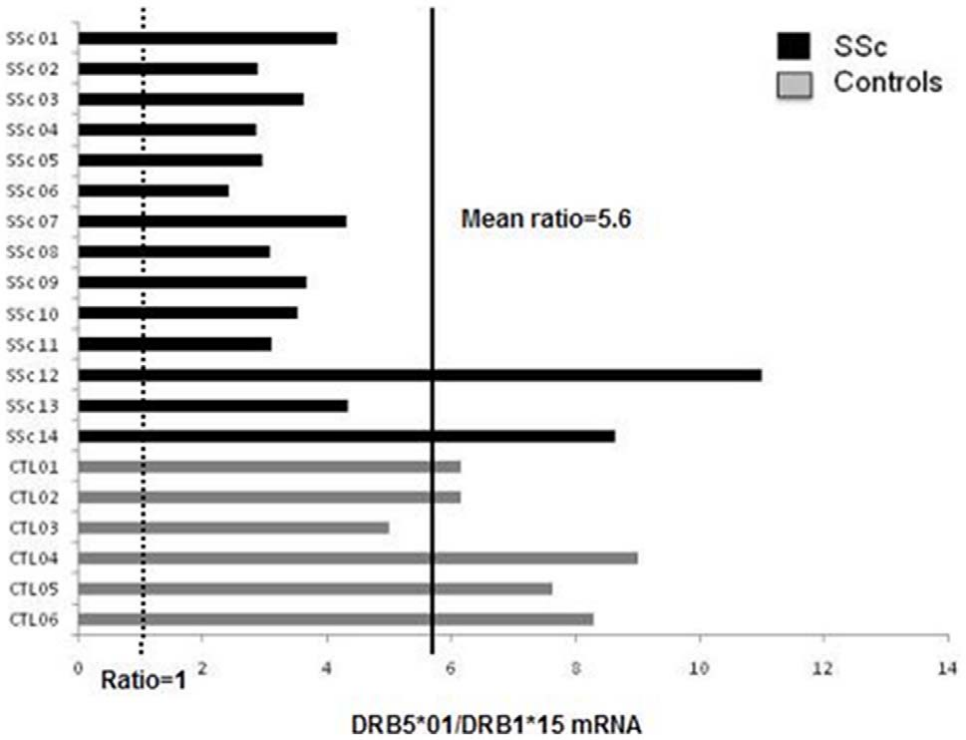
mRNA expression of DRB5*01 and DRB1*15 in patients with SSc and controls (CTL).

The FLEDR motif is expressed on the ß1 chain in some HLA-DR haplotypes (i.e. DR11), and on the ß5 chain on other HLA-DR haplotypes (i.e. DR15, see **Table 1**). Ratios of HLA-DRB5*01 (FLEDR^pos^)/HLA-DRB1*15 (FLEDR^neg^) mRNA expression were compared in 14 patients with SSc and 6 healthy controls **(** **Figure 1** **)**. Levels of ß5 mRNA (FLEDR^pos^) were systematically higher than levels of ß1 mRNA with a mean ratio of 5.6. This difference was observed in patients and controls.

As a control of our experiments and validation of our quantitative comparisons, we checked whether levels of ß1 mRNA were higher than ß3 mRNA in HLA-DR11 haplotypes as previously described. Indeed, analyses on 5 subjects (2 patients with SSc and 3 controls) confirmed an increased quantity of ß1 mRNA (FLEDR^pos^), with a mean ratio of 4.5, compared to ß3 mRNA (data not shown).

## Discussion

The most important genetic factors for scleroderma, as for many autoimmune diseases, are in the HLA locus. Indeed, results from worldwide cohorts of patients with SSc, going from early HLA allele frequency analyses to more recent Genome Wide Association studies, all show HLA association with SSc. Most studies have used the classification of diffuse or limited disease, while others, more recently, have analysed patients subsets classified according to their autoantibody status. Overall, the consensus is that HLA-DRB1*11∶04 is a risk factor in numerous Caucasian populations for diffuse SSc and presence of ATA [3], [5] and HLA-DRB1*15∶02 and DRB1*0802 in Asian populations [7]. Sequence identity on SSc-associated HLA-DRß chains conducted to a model of FLEDR shared epitope at amino acid positions 67 to 71 [16]. Similarly, SSc-associated HLA-DQß1 chains have a common ^71^TRAELDT^77^ sequence, which has been associated with patients with dcSSc and ATA. Although many studies have analysed HLA allele frequencies in several ethnic groups including North American Caucasians, Japanese and Choctaw Indians [2], no study has evaluated the strength of FLEDR motif compared to TRAELDT motif. Moreover, to our knowledge, this is the first study analysing HLA class II shared epitopes in French Caucasian patients with systemic sclerosis (SSc) stratified by clinical subsets *and* autoantibodies. We confirmed two previous findings in Japanese and Korean patients [16] and showed that FLEDR is the most prevalent shared epitope for the most severe type of SSc (dcSSc ATA+) in French Caucasians. We further showed that the association is linked to autoantibody production rather than clinical subtype. Finally by using standardized adjusted residual method we showed that having 2 doses of FLEDR is the higher risk to develop dcSSc with ATA.

Since TRAELDT association is weaker compared with FLEDR association, and since TRAELDT is often in linkage disequilibrium (LD) with FLEDR, one could think TRAELDT association is solely due to LD. However we showed that TRAELDT has its own contribution to disease susceptibility and autoantibody specificity. A tyrosine at position 30 (^30^Y), previously shown to reinforce the strength of the TRAELDT association with patients with ATA [9], added significance to the association but still remained lower than the FLEDR association. Similarly, on the DRß chain, by a novel approach which consists in subdividing into biologically relevant smaller sequence features and their variant types, Karp et al. showed that additional residual amino acids played a role in the risk to develop SSc [17]. Risk alleles had the sequence ^26^F-^28^D_^30^Y_^37^Y_^67^F/I_^70^D_^71^R_^86^V. However this additional effect of residual amino acids on DRß chain was not as obvious in our cohort as most double dose FLEDR shared epitopes were HLA-DRB1*11∶04/*15∶01 and HLA-DRB1*15∶01 does carry FLEDR but not the whole risk sequence described above (ie: ^86^V).

A parallel can be made between shared epitope in Rheumatoid Arthritis (RA) and in SSc. In patients with RA, the shared epitope ^70^QK/RRAA^74^ has a strong effect on the risk to develop Anti-Citrullinated Peptide Antibodies (ACPA) positive RA, whereas this association, although significant, is weaker in patients without ACPA [18]. This observation argues the admitted hypothesis that a particular HLA shared epitope presents particular auto-antigenic peptides triggering to a T cell helper response, which itself conducts to a particular auto-antibody production.

The FLEDR motif, by its position in the peptide binding groove, is determinant for efficient presentation of antigenic peptides to T cells. Interestingly, we showed that this motif would be overexpressed when carried by DRß5 chains in HLA-DR15 molecules, as well as when carried by DRß1 in HLA-DR11 molecules. Indeed, our results, very similar to Prat et al. recently found in patients with multiple sclerosis and controls [14], showed a 5 fold increase of ß5 chain at mRNA level. Prat et al. further showed that this mRNA increase correlated with a two-fold increase at protein level. ß5 chains might be then sufficiently expressed at cell surface to combine with the DRß chain to form additional DR molecules on the cell surface and be involved in antigen presentation [14]. These “accessory chains” serve to extend and complement the peptide repertoire of DRB1 in antigen presentation [19].

In the current study, not only we confirmed that some HLA-DRB1 and DQB1 alleles are highly associated with the production of ATA, but for the first time we statistically evaluated the strength of each HLA allele common motives. FLEDR is the main presenting motif involved in ATA production. Knowing better motives involved in peptide and autoantibody production could allow developing blocking therapies to prevent ATA production, a hallmark of higher risk for severe organ involvement, for internal malignancies and for reduced survival. Indeed, in a recent publication, by using an *in silico* molecular docking program to screen a large “druglike” chemical library, Michels et al. were able to find small molecules capable of occupying the pockets along the I-A^g7^ binding groove in the NOD mouse model of spontaneous autoimmune diabetes [20].

The focus of this paper has been the amino-acid sequence from position 67 to 71 encoded by *HLA-DRB* and the amino-acid sequence from position 71 to 77 encoded by *HLA-DQB1*, but classification of *HLA-DRB1* genotypes according to their risk should provide diagnostic markers for SSc. Indeed we found that HLA-DRB1*1104/DRB1*1501 was the most common FLEDR double dose genotype among patients with ATA and was rarely seen in healthy controls. This highlights a synergistic effect of different alleles from each haplotype. Double dose of shared epitope but also compound heterozygosity, may confer a higher risk to disease as it has been shown in rheumatoid arthritis, type 1 diabetes, celiac disease and systemic lupus erythematosus suggesting a common autoimmune pathway [19], [21], [22], [23], [24].

Future larger studies should also focus on classification by HLA genotypes at risk for SSc to provide help in clinical practice for a disease still difficult to diagnose.

## Supporting Information

Table S1 **HLA-DRB1 allele frequencies in patients with SSc divided by clinical subtypes and compared with healthy controls.** ^a^ Odds ratios (OR) and confidence intervals [CI] are given only for HLA-DRB1 allele frequencies statistically higher (susceptibility alleles) or statistically lower (protective alleles) in patients compared with controls. ^b^ Otherwise statistics are noted as non-significant (ns). ^c^ p<0.05 after correction for multiple comparisons.
(DOCX)

Table S2 **HLA-DQB1 allele’s frequencies in patients with SSc divided by clinical subtypes and compared with healthy controls.** ^a^ Odds ratios (OR) and confidence intervals [CI] are given only for HLA-DRB1 allele frequencies statistically higher (susceptibility alleles) or statistically lower (protective alleles) in patients compared with controls. ^b^ Otherwise statistics are noted as non-significant (ns). ^c^ p<0.05 after correction for multiple comparisons.
(DOCX)

Table S3 **HLA-DRB1 allele frequencies in patients with SSc divided by autoantibodies status and compared with healthy controls.** ^a^ Odds ratios (OR) and confidence intervals [CI] are given only for HLA-DRB1 allele frequencies statistically higher (susceptibility alleles) or statistically lower (protective alleles) in patients compared with controls. ^b^ Otherwise statistics are noted as non-significant (ns). ^c^ p<0.05 after correction for multiple comparisons.
(DOCX)

Table S4 **HLA-DQB1 allele’s frequencies in patients with SSc divided by autoantibodies status and compared with healthy controls.** ^a^ Odds ratios (OR) and confidence intervals [CI] are given only for HLA-DRB1 allele frequencies statistically higher (susceptibility alleles) or statistically lower (protective alleles) in patients compared with controls. ^b^ Otherwise statistics are noted as non-significant (ns). ^c^ p<0.05 after correction for multiple comparisons.
(DOCX)

Table S5 **FLEDR and TRAELDT haplotype analyses in patients with SSc classified by clinical subgroups.** Haplotypes FLEDR^0^ -TRAELDT^1/2^ in dcSSc/Haplotypes FLEDR^0^–TRAELD^1/2^ in healthy: χ^2^ = 12.9, p = 0.0003
(DOCX)

Table S6 **FLEDR and TRAELDT haplotype analyses in patients with SSc classified by autoantibody status.** Haplotypes FLEDR^0^ -TRAELDT^1/2^ in SSc ATApos/Haplotypes FLEDR0 -TRAELDT^1/2^ in healthy: χ^2^ = 19.4, p = 0.000013
(DOCX)
