# Supplementary material for: Comparing HLA Shared Epitopes in French Caucasian Patients with Scleroderma
Source: PLoS One. 2012 May 15;7(5):e36870. doi: 10.1371/journal.pone.0036870 (PMC3352938; doi:10.1371/journal.pone.0036870)
Supplement: Table S1 — HLA-DRB1 allele frequencies in patients with SSc divided by clinical subtypes and compared with healthy controls. a Odds ratios (OR) and confidence intervals [CI] are given only for HLA-DRB1 allele frequencies statistically higher (susceptibility alleles) or statistically lower (protective alleles) in patients compared with controls. b Otherwise statistics are noted as non-significant (ns). c p<0.05 after correction for multiple comparisons. (DOCX) [file pone.0036870.s001.docx]

| **HLA-DRB1** | | **Healthy ctrls** | | **DcSSc** | | | | **LcSSc** | | | |
| --- | --- | --- | --- | --- | --- | --- | --- | --- | --- | --- | --- |
|  | | **N=468** | | **N=94** | | | | **N=188** | | | |
| **generic** | **allelic** | **N ^all.^** | ***Freq. %*** | **N ^all.^** | ***Freq. %*** | ***OR [CI]^a^*** | ***P value*** | **N ^all.^** | ***Freq. %*** | ***OR [CI]*** | ***P value*** |
|  |  |  |  |  |  |  |  |  |  |  |  |
| ***01** |  | **84** | ***9.0*** | **8** | ***4.3*** | *0.45 [0.21-0.95]* | *0.037* | **53** | ***14.1*** | *1.66 [1.15-2.4]* | *0.006* |
| ***15** |  | **98** | ***10.5*** | **32** | ***17.0*** | *1.75 [1.13-2.7]* | *0.010* | **44** | ***11.7*** |  | *ns^b^* |
|  | *15:xx* | *1* |  | *2* |  |  |  | *0* |  |  |  |
|  | *15:01* | *88* |  | *27* |  |  |  | *43* |  |  |  |
|  | *15:02* | *9* |  | *2* |  |  |  | *1* |  |  |  |
|  | *15:03* | *0* |  | *0* |  |  |  | *0* |  |  |  |
|  | *15:04* | *0* |  | *1* |  |  |  | *0* |  |  |  |
| ***16** |  | **19** | ***2.0*** | **6** | ***3.2*** |  | *ns* | **14** | ***3.7*** |  | *ns* |
| ***03** |  | **101** | ***10.8*** | **20** | ***10.6*** |  | *ns* | **39** | ***10.4*** |  | *ns* |
| ***04** |  | **137** | ***14.6*** | **17** | ***9.0*** | *0.58 [0.34-0.99]* | *0.042* | **65** | ***17.3*** |  | *ns* |
| ***11** |  | **148** | ***15.8*** | **53** | ***28.2*** | ***2.03 [1.41-2.92]*** | ***0.0001****^c^* | **52** | ***13.8*** |  | *ns* |
|  | *11:01/4* | *0* |  | *1* |  |  |  | *0* |  |  |  |
|  | *11:01* | *86* |  | *15* |  |  |  | *22* |  |  |  |
|  | *11:02* | *7* |  | *5* |  |  |  | *3* |  |  |  |
|  | *11:03* | *15* |  | *3* |  |  |  | *7* |  |  |  |
|  | *11:04* | *39* |  | *29* |  |  |  | *20* |  |  |  |
|  | *11:45* | *1* |  | *0* |  |  |  | *0* |  |  |  |
| ***12** |  | **16** | ***1.7*** | **1** | ***0.5*** |  | *ns* | **3** | ***0.8*** |  | *ns* |
| ***13** |  | **121** | ***12.9*** | **16** | ***8.5*** |  | *ns* | **30** | ***8.0*** | *0.58 [0.38-0.88]* | *0.011* |
| ***14** |  | **44** | ***4.7*** | **5** | ***2.7*** |  | *ns* | **12** | ***3.2*** |  | *ns* |
| ***07** |  | **112** | ***12.0*** | **18** | ***9.6*** |  | *ns* | **28** | ***7.5*** | *0.59 [0.38-0.91]* | *0.017* |
| ***08** |  | **32** | ***3.4*** | **11** | ***5.9*** |  | *ns* | **26** | ***6.9*** | *2.1 [1.23-3.57]* | *0.0053* |
|  | *08:01/2* | *1* |  | *0* |  |  |  | *0* |  |  |  |
|  | *08:01* | *22* |  | *6* |  |  |  | *20* |  |  |  |
|  | *08:02* | *0* |  | *2* |  |  |  | *1* |  |  |  |
|  | *08:03* | *4* |  | *1* |  |  |  | *1* |  |  |  |
|  | *08:04* | *4* |  | *1* |  |  |  | *4* |  |  |  |
|  | *08:06* | *1* |  | *1* |  |  |  | *0* |  |  |  |
| ***09** |  | **11** | ***1.2*** | **0** | ***0.0*** |  | *ns* | **4** | ***1.1*** |  | *ns* |
| ***10** |  | **13** | ***1.4*** | **1** | ***0.5*** |  | *ns* | **6** | ***1.6*** |  | *ns* |
| **Total # alleles** | | **936** |  | **184** |  |  |  | **276** |  |  |  |

^a^ Odds ratios (OR) and confidence intervals [CI] are given only for HLA-DRB1 allele frequencies statistically higher (susceptibility alleles) or statistically lower (protective alleles) in patients compared with controls. ^b^ Otherwise statistics are noted as non-significant (ns). ^c^ p< 0.05 after correction for multiple comparisons.

**Table S1**- HLA-DRB1 allele frequencies in patients with SSc divided by clinical subtypes and compared with healthy controls
