# Supplementary material for: Comparing HLA Shared Epitopes in French Caucasian Patients with Scleroderma
Source: PLoS One. 2012 May 15;7(5):e36870. doi: 10.1371/journal.pone.0036870 (PMC3352938; doi:10.1371/journal.pone.0036870)
Supplement: Table S2 — HLA-DQB1 allele’s frequencies in patients with SSc divided by clinical subtypes and compared with healthy controls. a Odds ratios (OR) and confidence intervals [CI] are given only for HLA-DRB1 allele frequencies statistically higher (susceptibility alleles) or statistically lower (protective alleles) in patients compared with controls. b Otherwise statistics are noted as non-significant (ns). c p<0.05 after correction for multiple comparisons. (DOCX) [file pone.0036870.s002.docx]

| **HLA-DQB1** | **Healthy ctrls** | | **DcSSc** | | | | | **LcSSc** | | | |
| --- | --- | --- | --- | --- | --- | --- | --- | --- | --- | --- | --- |
| **generic** | **N=467** |  | **N=94** |  |  |  | | **N=186** |  |  |  |
|  | **N^all^.** | ***Freq. %*** | **N^all^.** | ***Freq. %*** | ***OR [CI]*** | ***P value*** | | **N^all^.** | ***Freq. %*** | ***OR [CI]*** | ***P value*** |
| ***02** | **187** | ***20.0*** | **30** | ***16.0*** |  | | *ns^b^* | **54** | ***14.5*** | *0.68 [0.49-0.95]* | *0.022* |
| ***03** | **346** | ***37.0*** | **83** | ***44.2*** |  | | *ns* | **138** | ***37.1*** |  | *ns* |
| ***04** | **29** | ***3.1*** | **8** | ***4.3*** |  | | *ns* | **24** | ***6.45*** | ***2.16 [1.24-3.76]*** | ***0.006^c^*** |
| ***05** | **166** | ***17.7*** | **20** | ***10.6*** | *0.55 [0.34-0.9]* | | *0.02* | **87** | ***23.4*** | *1.42 [1.06-1.9]* | *0.02* |
| ***06** | **208** | ***22.2*** | **47** | ***25.0*** |  | | *ns* | **69** | ***18.5*** |  | *ns* |
| **Total # alleles** | **934** |  | **188** |  |  | |  | **372** |  |  |  |

^a^ Odds ratios (OR) and confidence intervals [CI] are given only for HLA-DRB1 allele frequencies statistically higher (susceptibility alleles) or statistically lower (protective alleles) in patients compared with controls. ^b^ Otherwise statistics are noted as non-significant (ns). ^c^ p < 0.05 after correction for multiple comparisons.

**Table S2**- HLA-DQB1 allele’s frequencies in patients with SSc divided by clinical subtypes and compared with healthy controls
