# Supplementary material for: Comparing HLA Shared Epitopes in French Caucasian Patients with Scleroderma
Source: PLoS One. 2012 May 15;7(5):e36870. doi: 10.1371/journal.pone.0036870 (PMC3352938; doi:10.1371/journal.pone.0036870)
Supplement: Table S3 — HLA-DRB1 allele frequencies in patients with SSc divided by autoantibodies status and compared with healthy controls. a Odds ratios (OR) and confidence intervals [CI] are given only for HLA-DRB1 allele frequencies statistically higher (susceptibility alleles) or statistically lower (protective alleles) in patients compared with controls. b Otherwise statistics are noted as non-significant (ns). c p<0.05 after correction for multiple comparisons. (DOCX) [file pone.0036870.s003.docx]

| **HLA-DRB1** | | **Healthy ctrls,** | | **SSc Ab neg** | | | | **SSc ACA pos** | | | | **SSc ATA pos** | | | |
| --- | --- | --- | --- | --- | --- | --- | --- | --- | --- | --- | --- | --- | --- | --- | --- |
|  |  | **N=468** | | **N=80** | | | | **N=89** | | | | **N=74** | | | |
| **generic** | **allelic** | **N^all.^** | ***Freq %*** | **N^all.^** | ***Freq%*** | ***OR[CI]*** | ***P value*** | **N^all.^** | ***Freq%*** | ***OR [CI]*** | ***P value*** | **N^all.^** | ***Freq%*** | ***OR [CI]*** | ***P value*** |
| ***01** |  | **84** | ***9.0*** | **15** | ***9.4*** |  | *ns^b^* | **31** | ***17.4*** | ***2.14 [1.37-3.35]*** | ***0.0007^c^*** | **6** | ***4.1*** | *0.43 [0.18-1.0]* | *0.044* |
| ***15** |  | **98** | ***10.5*** | **22** | ***13.8*** |  | *ns* | **12** | ***6.7*** |  | *ns* | **31** | ***21.0*** | ***2.27 [1.45-3.55]*** | ***0.0003^c^*** |
|  | *15:xx* | *1* |  | *0* |  |  |  | *0* |  |  |  | *2* |  |  |  |
|  | *15:01* | *88* |  | *20* |  |  |  | *11* |  |  |  | *28* |  |  |  |
|  | *15:02* | *9* |  | *2* |  |  |  | *1* |  |  |  | *0* |  |  |  |
|  | *15:03* | *0* |  | *0* |  |  |  | *0* |  |  |  | *0* |  |  |  |
|  | *15:04* | *0* |  | *0* |  |  |  | *0* |  |  |  | *1* |  |  |  |
| ***16** |  | **19** | ***2.0*** | **6** | ***3.8*** |  | *ns* | **4** | ***2.3*** |  | *ns* | **4** | ***2.7*** |  |  |
| ***03** |  | **101** | ***10.8*** | **19** | ***11.9*** |  | *ns* | **22** | ***12.4*** |  | *ns* | **12** | ***8.1*** |  |  |
| ***04** |  | **137** | ***14.6*** | **24** | ***15.0*** |  | *ns* | **33** | ***18.5*** |  | *ns* | **10** | ***6.8*** | *0.42 [0.22-0.82]* | *0.009* |
| ***11** |  | **148** | ***15.8*** | **20** | ***12.5*** |  | *ns* | **26** | ***14.6*** |  | *ns* | **51** | ***34.5*** | ***2.8 [1.91-4.1]*** | ***<10^-6c^*** |
|  | *11:01/4* | *0* |  | *1* |  |  |  | *0* |  |  |  | *0* |  |  |  |
|  | *11:01* | *86* |  | *11* |  |  |  | *14* |  |  |  | *10* |  |  |  |
|  | *11:02* | *7* |  | *3* |  |  |  | *0* |  |  |  | *4* |  |  |  |
|  | *11:03* | *15* |  | *1* |  |  |  | *4* |  |  |  | *3* |  |  |  |
|  | *11:04* | *39* |  | *4* |  |  |  | *8* |  |  |  | *34* |  |  |  |
|  | *11:45* | *1* |  | *0* |  |  |  | *0* |  |  |  | *0* |  |  |  |
| ***12** |  | **16** | ***1.7*** | **0** | ***0.0*** |  | *ns* | **2** | ***1.1*** |  | *ns* | **1** | ***0.7*** |  |  |
| ***13** |  | **121** | ***12.9*** | **19** | ***11.9*** |  | *ns* | **10** | ***5.6*** | *0.4 [0.21-0.78]* | *0.005* | **9** | ***6.1*** | *0.44 [0.22-0.89]* | *0.017* |
| ***14** |  | **44** | ***4.7*** | **4** | ***2.5*** |  | *ns* | **8** | ***4.5*** |  | *ns* | **3** | ***2.0*** |  |  |
| ***07** |  | **112** | ***12.0*** | **17** | ***10.6*** |  | *ns* | **9** | ***5.1*** | *0.39 [0.19-0.78]* | *0.006* | **14** | ***9.5*** |  |  |
| ***08** |  | **32** | ***3.4*** | **9** | ***5.6*** |  | *ns* | **15** | ***8.4*** | ***2.6 [1.38-4.91]*** | ***0.002^c^*** | **7** | ***4.7*** |  |  |
|  | *08:01/2* | *1* |  | *0* |  |  |  | *0* |  |  |  | 0 |  |  |  |
|  | *08:01* | *22* |  | *5* |  |  |  | *11* |  |  |  | 4 |  |  |  |
|  | *08:02* | *0* |  | *1* |  |  |  | *1* |  |  |  | 1 |  |  |  |
|  | *08:03* | *4* |  | *0* |  |  |  | *0* |  |  |  | 1 |  |  |  |
|  | *08:04* | *4* |  | *3* |  |  |  | *3* |  |  |  | 0 |  |  |  |
|  | *08:06* | *1* |  | *0* |  |  |  | *0* |  |  |  | 1 |  |  |  |
| ***09** |  | **11** | ***1.2*** | **2** | ***1.3*** |  | *ns* | **2** | ***1.1*** |  | *ns* | **0** | ***0*** |  |  |
| ***10** |  | **13** | ***1.4*** | **3** | ***1.9*** |  | *ns* | **4** | ***2.3*** |  | *ns* | **0** | ***0*** |  |  |
| **Total # alleles** | | **936** |  | **160** |  |  |  | **178** |  |  |  | **148** |  |  |  |

^a^ Odds ratios (OR) and confidence intervals [CI] are given only for HLA-DRB1 allele frequencies statistically higher (susceptibility alleles) or statistically lower (protective alleles) in patients compared with controls. ^b^ Otherwise statistics are noted as non-significant (ns). ^c^ p< 0.05 after correction for multiple comparisons.

**Table S3**- HLA-DRB1 allele frequencies in patients with SSc divided by autoantibodies status and compared with healthy controls.
