# Supplementary material for: Comparing HLA Shared Epitopes in French Caucasian Patients with Scleroderma
Source: PLoS One. 2012 May 15;7(5):e36870. doi: 10.1371/journal.pone.0036870 (PMC3352938; doi:10.1371/journal.pone.0036870)
Supplement: Table S4 — HLA-DQB1 allele’s frequencies in patients with SSc divided by autoantibodies status and compared with healthy controls. a Odds ratios (OR) and confidence intervals [CI] are given only for HLA-DRB1 allele frequencies statistically higher (susceptibility alleles) or statistically lower (protective alleles) in patients compared with controls. b Otherwise statistics are noted as non-significant (ns). c p<0.05 after correction for multiple comparisons. (DOCX) [file pone.0036870.s004.docx]

| **HLA-DQB** | **Healthy ctrls** | | **SSc Ab neg** | | | | **SSc ACA pos** | | | | **SSc ATA pos** | | | |
| --- | --- | --- | --- | --- | --- | --- | --- | --- | --- | --- | --- | --- | --- | --- |
| **generic** | **N=467** | | **N=78** | | | | **N=88** |  |  |  | **N=75** |  |  |  |
|  | **N^all.^** | **Freq %** | **N^all.^** | **Freq %** | **OR [CI]** | **P value** | **N^all.^** | **Freq%** | **OR [CI]** | **P value** | **N^all.^** | **Freq. %** | **OR [CI]** | **P value** |
|  |  |  |  |  |  |  |  |  |  |  |  |  |  |  |
| ***02** | **186** | ***20.0*** | **29** | ***18.6*** |  | *ns* | **26** | ***14.8*** |  | *ns* | **18** | ***12.0*** | *0.54 [0.32-0.91]* | *0.02* |
| ***03** | **346** | ***37.0*** | **53** | ***34.0*** |  | *ns* | **66** | ***37.5*** |  | *ns* | **73** | ***48.7*** | ***1.61 [1.14-2.28]*** | ***0.007*** |
| ***04** | **29** | ***3.1*** | **8** | ***5.1*** |  | *ns* | **15** | ***8.5*** | ***2.91 [1.53-5.55]*** | ***0.0007*** | **5** | ***3.3*** |  | *ns* |
| ***05** | **166** | ***17.7*** | **28** | ***18.0*** |  | *ns* | **49** | ***27.8*** | ***1.78 [1.23-2.58]*** | ***0.002*** | **15** | ***10.0*** | *0.51 [0.29-0.89]* | *0.02* |
| ***06** | **207** | ***22.2*** | **38** | ***24.4*** |  | *ns* | **20** | ***11.4*** | ***0.45 [0.28-0.73]*** | ***0.001*** | **39** | ***26.0*** |  | *ns* |
| **Total # alleles** | **934** |  | **156** |  |  |  | **176** |  |  |  | **150** |  |  |  |

^a^ Odds ratios (OR) and confidence intervals [CI] are given only for HLA-DRB1 allele frequencies statistically higher (susceptibility alleles) or statistically lower (protective alleles) in patients compared with controls. ^b^ Otherwise statistics are noted as non-significant (ns). ^c^ p< 0.05 after correction for multiple comparisons.

**Table S4**- HLA-DQB1 allele’s frequencies in patients with SSc divided by autoantibodies status and compared with healthy controls.
