# Supplementary material for: Comparing HLA Shared Epitopes in French Caucasian Patients with Scleroderma
Source: PLoS One. 2012 May 15;7(5):e36870. doi: 10.1371/journal.pone.0036870 (PMC3352938; doi:10.1371/journal.pone.0036870)
Supplement: Table S5 — FLEDR and TRAELDT haplotype analyses in patients with SSc classified by clinical subgroups. Haplotypes FLEDR0 -TRAELDT1/2 in dcSSc/Haplotypes FLEDR0–TRAELD1/2 in healthy: χ2 = 12.9, p = 0.0003 (DOCX) [file pone.0036870.s005.docx]

|  |  | **Healthy controls** | | | | | **DcSSc** | | | | | **LcSSc** | | | | |
| --- | --- | --- | --- | --- | --- | --- | --- | --- | --- | --- | --- | --- | --- | --- | --- | --- |
|  |  | **N=468** | | | | | **N=94** | | | | | **N=188** | | | | |
|  |  | **TRAELDT doses** | | | | | | | | | | | | | | |
|  |  | **2** | **1** | **0** | **Total** | **%** | **2** | **1** | **0** | **Total** | **%** | **2** | **1** | **0** | **Total** | **%** |
| **FLEDR doses** | **2** | 27 | 14 | 1 | **42** | ***9.0*** | 17 | 4 | 0 | **21** | ***22.3*** | 7 | 11 | 3 | **21** | ***11.2*** |
|  | **1** | 62 | 110 | 12 | **184** | ***39.3*** | 17 | 29 | 4 | **50** | ***53.2*** | 31 | 42 | 11 | **84** | ***44.7*** |
|  | **0** | 66 | 120 | 56 | **242** | ***51.7*** | 10 | 9 | 4 | **23** | ***24.5*** | 16 | 48 | 19 | **83** | ***44.1*** |
|  | **Total** | **155** | **244** | **69** | **468** | ***100.0*** | **44** | **42** | **8** | **94** | ***100.0*** | **54** | **101** | **33** | **188** | ***100.0*** |
|  | **%** | ***33.1*** | ***52.1*** | ***14.7*** | ***100.0*** |  | ***46.8*** | ***44.7*** | ***8.5*** | ***100.0*** |  | ***28.7*** | ***53.7*** | ***17.6*** | ***100.0*** |  |

Haplotypes FLEDR^0^ -TRAELDT^1 /2^ in dcSSc / Haplotypes FLEDR^0^ –TRAELD^1/2^ in healthy: χ²= 12.9, p=0.0003

**Table S5**- FLEDR and TRAELDT haplotype analyses in patients with SSc classified by clinical subgroups
