# Supplementary material for: Comparing HLA Shared Epitopes in French Caucasian Patients with Scleroderma
Source: PLoS One. 2012 May 15;7(5):e36870. doi: 10.1371/journal.pone.0036870 (PMC3352938; doi:10.1371/journal.pone.0036870)
Supplement: Table S6 — FLEDR and TRAELDT haplotype analyses in patients with SSc classified by autoantibody status. Haplotypes FLEDR0 -TRAELDT1/2 in SSc ATApos/Haplotypes FLEDR0 -TRAELDT1/2 in healthy: χ2 = 19.4, p = 0.000013 (DOCX) [file pone.0036870.s006.docx]

|  |  | **SSc Ab neg** | | | | | | **SSc ACA pos** | | | | | | **SSc ATA pos** | | | | | |
| --- | --- | --- | --- | --- | --- | --- | --- | --- | --- | --- | --- | --- | --- | --- | --- | --- | --- | --- | --- |
|  |  | **N=80** | | | | | | **N=89** | | | | | | **N= 74** | | | | | |
|  |  | **TRAELDT doses** | | | | | | | | | | | | | | | | | |
|  |  | **2** | **1** | **0** | **Total** | | **%** | **2** | **1** | **0** | **Total** | | **%** | **2** | **1** | **0** | **Total** | | **%** |
| **FLEDR doses** | **2** | 3 | 4 | 0 | **7** | ***8.8*** | | 1 | 4 | 3 | **8** | ***9.0*** | | 17 | 4 | 0 | **21** | ***28.4*** | |
|  | **1** | 14 | 20 | 7 | **41** | ***51.3*** | | 11 | 21 | 5 | **37** | ***41.6*** | | 19 | 22 | 1 | **42** | ***56.8*** | |
|  | **0** | 12 | 12 | 8 | **32** | ***40.0*** | | 6 | 25 | 13 | **44** | ***49.4*** | | 3 | 7 | 1 | **11** | ***14.9*** | |
|  | **Total** | **29** | **36** | **15** | **80** | ***100.0*** | | **18** | **50** | **21** | **89** | ***100.0*** | | **39** | **33** | **2** | **74** | ***100.0*** | |
|  | **%** | ***36.3*** | ***45.0*** | ***18.8*** | ***100.0*** |  | | ***20.2*** | ***56.2*** | ***23.6*** | ***100.0*** |  | | ***52.7*** | ***44.6*** | ***2.7*** | ***100.0*** |  | |

Haplotypes FLEDR^0^ -TRAELDT^1 / 2^ in SSc ATApos / Haplotypes FLEDR^0^ -TRAELDT^1 /2^ in healthy: χ²= 19.4, p=0.000013

**Table S6**- FLEDR and TRAELDT haplotype analyses in patients with SSc classified by autoantibody status
